# The Agent Brain: A Review of Non-invasive Brain Stimulation Studies on Sensing Agency

**DOI:** 10.3389/fnbeh.2017.00229

**Published:** 2017-11-20

**Authors:** Davide Crivelli, Michela Balconi

**Affiliations:** ^1^Research Unit in Affective and Social Neuroscience, Catholic University of the Sacred Heart, Milan, Italy; ^2^Department of Psychology, Catholic University of the Sacred Heart, Milan, Italy

**Keywords:** sense of agency, NIBS, TMS, tES, tDCS, social understanding, body ownership

## Abstract

According to philosophy of mind and neuroscientific models, the sense of agency can be defined as the sense that I am the one that is generating an action and causing its effects. Such ability to sense ourselves as causal agents is critical for the definition of intentional behavior and is a primary root for human interaction skills. The present mini-review aims at discussing evidences from non-invasive brain stimulation (NIBS) studies targeting functional correlates of different aspects of agency and evidences on the way stimulation techniques affect such core feature of human subjective experience. Clinical and brain imaging studies helped in defining a neural network mediating agency-related processes, which includes the dorsolateral prefrontal cortex (dlPFC), the cingulate cortex (CC), the supplementary and pre-supplementary motor areas (SMA and pre-SMA), the posterior parietal cortex (PPC) and its inferior regions and the cerebellum. However, while the plurality of those structures mirrors the complexity of the phenomenon, their actual roles with respect to different components of the experience of agency have been primarily explored via correlational techniques, without a clear evidence about their causal significance with respect to the integration of sensorimotor information, intentionalization, and action monitoring processes. Therefore, insights into the specific causal role of different cortical structures can be specified by using NIBS techniques, in order to provide improved understanding into the bases of our ability vs. inability to properly act in complex social contexts.

## Introduction

When we do act and observe the consequences of our action, we tend to be sure that we were the ones who were acting. That kind of awareness mirrors the common experience of being an agent. That feeling—together with unified subjective experiences and with the first-person point-of-view from where we perceive and qualify those experiences—is what defines human self-consciousness (Metzinger, 2003). The feeling of being agents able to influence our environment is one of the main roots that sustain and support that being-in-the-world. Our representations of the context, of ourselves and of their relationship play, in turn, a fundamental role for the definition of our agentive stance (Haggard, 2008, 2017; Synofzik et al., 2008b; Carruthers, 2009; Balconi, 2010a,b; Frith, 2013; Smith, 2016).

The richness of debates on the experience of being an agent and its physiological, other than phenomenological, correlates have been fuelled by a remarkable set of evidences obtained via neuroscientific techniques such as neuroimaging, electrophysiology and brain stimulation. The majority of data concerning neural structures supporting the components of the experience of agency have been, to date, primarily explored via correlational techniques, such as functional imaging (David, 2010). Differently, non-invasive brain stimulation (NIBS) techniques can provide insight concerning the specific causal role of different cortical structures with regard to a mental process or a specific function, and then foster our understanding of the bases of our ability vs. inability to sense and judge the authorship of our behavior and its outcomes. After an introduction on core aspects of the construct of agency and on the neural network that is thought to let us experience agency, we will critically compare the potential of correlational and different brain stimulation techniques as investigation tools. Finally, we will focus on NIBS studies targeting functional correlates of different aspects of agency and evidences on the way stimulation techniques affect such core feature of human subjective experience.

## Agency as a Core Feature of Human Experience

Here, we define the sense of agency as the sense that I am the one that is generating an action, would it be physical or mental, and causing its effects (Gallagher, 2000). Such theoretical construct is deeply bound to the one of sense of ownership, which can, instead, be defined as the sense that I am the one who is undergoing an experience. Nonetheless, the sense of agency and the sense of ownership can be experienced as separate entities and can be independently affected by experimental manipulation and pathology. Neurological and psychiatric disorders, such as the alien hand syndrome and schizophrenia, can be, for example, associated to ownership and agency disruption (Jahanshahi, 1998; Bottini et al., 2002; Synofzik et al., 2008b; Jardri et al., 2009; Jeannerod, 2009; Synofzik and Voss, 2010).

Human experience is shaped by the potential to be aware of the causal power we can exert on the external world and by self-attribution of a primary agentive role with regard to our actions (from its simpler manifestations like basic goal-directed motor acts to its more complex manifestations like moral reasoning and reflective judgments concerning responsibilities). Accordingly, modern phenomenology posits that experience is grounded “in neural activity in embodied action in appropriate surroundings” (Smith, 2016). Assuming that agency is core feature for human experience (Metzinger, 2003; Haggard, 2008; Smith, 2016), it has also been suggested that in the very moment we sense ourselves as intentional agents and we consciously self-attribute an agentive stance, we have to assume that other individuals might share the same capability and a similar stance. Further, besides being able to sense and judge the authorship of our behavior and its outcomes, we also need to be able to apply those detection and attribution processes to other potential inter-agents. Those skills are critical to adequately act in a social context and to intentionally exert control on a social interactions (see Crivelli and Balconi, 2010, 2015; Crivelli, 2016), would them be dyadic or collective—such as in team sports. Again they might act as the soil where higher social understanding skills such as inter-personal co-regulation and mind-reading sink their roots (Gallagher and Meltzoff, 1996; Saxe, 2006).

Agency is, then, a complex phenomenon influenced by intention, goals and desires but also grounded on somatosensory signals and afferent sensory information flow. The integration of different levels is necessary for the rising of a complete experience. Body image and intentional structure merge to generate unified agents capable of perceiving, influencing, and exerting causal power on each other and on the environment, understood as a complex system including both objects to act with and subjects to interact with.

## The Agent Brain

**Figure 1:**
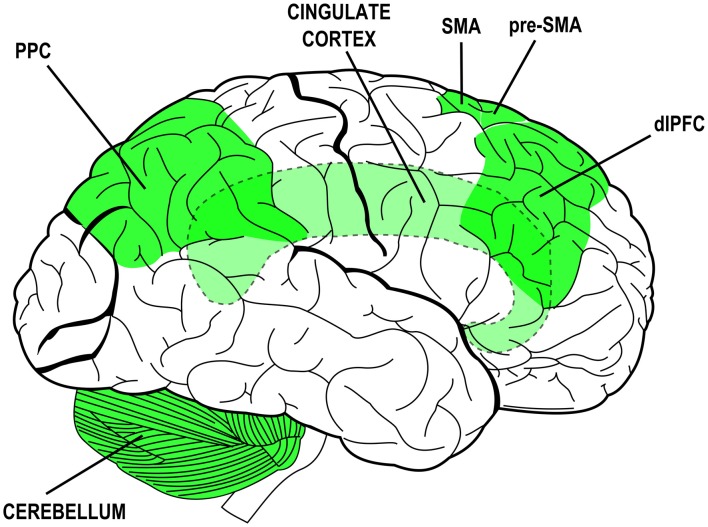
Brain structures associated to functions and processes subserving the experience of agency and their distribution over the brain (lateral view). Simplified schematic representation. Dashed outlines and lighter colors indicate subcortical or hidden structures. PPC, posterior parietal cortex; SMA, supplementary motor area; pre-SMA, pre-supplementary motor area; dlPFC, dorsolateral prefrontal cortex.

Many neural structures have been associated to specific attributes of agency experience or to specific steps of the process that leads to *sense* agency. The collection of neural structures that are thought to mediate agency-related processes is rather wide and includes: the dorsolateral prefrontal cortex (dlPFC), the cingulate cortex (CC), the supplementary and pre-supplementary motor areas (SMA and pre-SMA), the posterior parietal cortex (PPC) and its inferior regions and the cerebellum (Gehring et al., 1990; Lee et al., 1999; Blakemore et al., 2001; Chaminade and Decety, 2002; Cunnington et al., 2002; Farrer and Frith, 2002; Blakemore and Sirigu, 2003; Farrer et al., 2003; Lau et al., 2004, 2006; Synofzik et al., 2008a; Balconi and Crivelli, 2009, 2010a,b; Balconi and Scioli, 2012; Balconi et al., 2017). The plurality of those structures and their distribution over the whole brain (see Figure 1) likely mirrors the complexity of the phenomenon and the different methodological and experimental approaches devised to study its facets (for a review see also David, 2010). Again, they are due to the contribution of multiple mechanisms in the coupling of behavior with mental states and sensory effects. Those mechanisms—and the structures that subserve them—can be traced back to overarching functions: monitoring of sensorimotor congruence and multimodal integration, intentionalization (i.e., elaboration and implementation of intentions), action monitoring and ownership/agency attribution.

## Stimulating The Agent Brain: Non-invasive Brain Stimulation Evidences

Neuroimaging and electrophysiological methods can be both enlisted among the correlational techniques (Walsh and Cowey, 2000), which allow for qualifying and quantifying ongoing neural activity during implicit or explicit tasks to compare it with co-occurrent subjective experience, cognitive performances, or behavior. By superimposing and integrating those series of data, it is possible to draw conclusions on anatomical-functional correlates of investigated functions and processes by means of association. Conversely, non-invasive stimulation methods can be enlisted among interference or causal techniques, which grant the advantage of drawing conclusion on neural causation and on the effective role of neural structures in supporting or modulating a specific function or process (Woods et al., 2016). Indeed, NIBS can be used to perturb the ongoing activity of a target structure during implicit or explicit tasks and then observe the consequences of such perturbation on behavior and/or neural activity (e.g., by means of EEG). It is worth noting that conclusions that can be drawn thanks to NIBS studies also show fewer potential biases than those deriving from clinical lesion studies (Walsh and Cowey, 2000). Relevant for the present discussion, NIBS techniques then present notably greater cognitive resolution—defined as the ability to tell something new about brain processes and to answer a wide range of questions on cognitive functioning and its physiological correlates (Walsh and Pascual-Leone, 2003)—with respect to other investigation tools.

Within the panorama of NIBS, two main families of techniques have been used in research on agency: transcranial magnetic stimulations (TMS) and transcranial electrical stimulations (tES). Both TMS and tES inform us on brain functioning by interfering with neural activity, but they are thought to do so in different ways. In particular, TMS is deemed as a neurostimulation tool in that it is able to actually initiate action potentials in a nerve cell or axon by inducing non-invasively transient electrical currents at the level of cortical tissues. The mechanism of action grounds on the Faraday’s law of electromagnetic induction and stimulation is induced by brief magnetic pulses delivered through a coil. Instead, tES (with lowercase *t* so to refer to techniques using low-intensity electrical stimulation, instead of high-voltage TES) is deemed as a neuromodulation tool in that it is able to modulate spontaneous neuronal firing rates by causing small changes in the membrane potential of the nerve cells or axons (Woods et al., 2016). The mechanism of action has been linked to long-term potentiation/depression mechanisms, even though the integration of the stimulation effects with a second input (e.g., neural oscillatory activity and thalamo-cortical stimulation) is thought to be needed to induce such phenomena (Fritsch et al., 2010). TMS and tES also differ in terms of spatial and time resolution and in terms of usability and side effects. TMS, when used in single-pulse or paired-stimulation protocols, show greater time resolution than tES applications and can then be more aptly used to investigate mental chronometry. Again, while TMS applications allow for stimulating quite focal portions of the cortical tissues, tES is able to modulate only broader populations of neurons or to influence transmission of information in neural networks. At the same time, tES applications lead to fewer unwanted side effects and are less noisy than magnetic stimulations, thus exerting minimal disturbance during task execution. The one technique or the other has then been chosen in different studies depending on the research question and technical-methodological needs.

The few NIBS investigations on the neural processes and mechanisms that make us sense agency essentially focused on the role of parietal and prefrontal areas with regard to sensorimotor integration, to the link between intentions and behavior, and to ownership definition processes underlying agency attribution.

### NIBS, Sensorimotor Congruence and Agency

There are evidences that offline low-frequency repetitive TMS—a stimulation protocol thought to have inhibition effects on the activity of stimulated portions of cortical tissues—applied to the left PPC, lead to an impairment in assessing asynchrony between a movement and its visual feedback, specifically when the movement is active and voluntary (MacDonald and Paus, 2003). At the same time, online high-frequency repetitive TMS (10 Hz) applied to the right inferior parietal cortex proved to induce healthy participants to misperceive their agentive role during self-controlled movements and to experience them as being externally perturbed even if it was not the case (Ritterband-Rosenbaum et al., 2014).

The critical role of parietal structures for the integration and comparison of sensorimotor information flows to correctly attribute movements agency has been underlined even by using non-repetitive magnetic stimulation protocols. Preston and Newport (2008), indeed, observed that when the activity of right inferior parietal lobule (IPL) is temporarily and focally tampered, healthy participants tend to attribute agency to external sources, often erroneously, regardless of the fact that perceivable visual feedbacks are consistent with executed actions or not. Again, single-pulse TMS has been recently used to explore the role of left inferior parietal areas in prospective components of the sense of agency, linked to action selection and programming processes independently from action effects (Chambon et al., 2015). By combining TMS with subliminal priming of action selection, the authors showed that the perturbation of the activity of left inferior parietal regions at the time of action selection and execution reduces participants’ perceived control over subsequent action effects. The importance of parietal/pre-motor connections to solve sensorimotor conflicts and finalize agency-attribution processes has been underlined even by Karabanov et al. (2017) by using a paired-pulse stimulation protocol. The authors, by implementing a motor version of the rubber hand illusion, observed similar inhibitory parietal-to-motor connectivity at rest and during illusory conditions inducing both sense of agency and ownership.

### NIBS, Action Control and Agency

To our best knowledge, the few NIBS investigations of the causal link between prefrontal action control and intentionalization processes and agency, used electrical stimulation techniques to modulate cortical activity and intentional binding to implicitly measure changes of the sense of agency. The intentional binding effect has to do with the reduction of perceived time interval between a voluntary action and its external sensory consequence when we do sense to be the primary agent of such action (Tsakiris and Haggard, 2003).

In particular, a recent meta-analysis of previously performed studies, highlighted that anodal stimulation—a continuous current electrical stimulation protocol thought to enhance cortical excitability and responsivity—applied to left dlPFC increases the intentional binding effect and, thus, implicit sense of agency, but only when people are free to act as they prefer (Khalighinejad et al., 2016). Again, even pre-SMA seems to contribute to implicit feeling of being an agent, thought results still need to be examined in depth. Indeed, Cavazzana et al. (2015) showed that both anodal and cathodal stimulation—a stimulation protocol thought to lower cortical excitability and responsivity—of pre-SMA areas leads to a relevant reduction of the intentional binding effect, hinting at the contribution of a medial frontal–prefrontal network for awareness and control of voluntary action. Finally, the perception of the temporal relationship between voluntary action and its perceivable consequences seems to be negatively affected by anodal stimulation of the left angular gyrus, critical for explicit agency judgments (Khalighinejad and Haggard, 2015).

A few final works did report studies where NIBS has not been properly used as an investigation technique but as a tool to create *ad hoc* experimental conditions. Namely, in the next two examples, TMS has been used to make participants move involuntarily and to deliver a complex action feedback. In the first case, the phenomenon of sensory attenuation linked to self-initiated actions and its contribution to differentiation of one’s own from others’ actions have been investigated by comparing neural responses to perceivable consequences (sounds) of voluntary and TMS-induced actions (Timm et al., 2014). The second case has to do with one of the first empirical investigation of the phenomenon of intentional binding. TMS has been used to deliver a complex somatosensory feedback (a muscular twitch) as a consequence of voluntary (self-initiated finger movements) and involuntary (passive finger movements caused by an external device) actions (Tsakiris and Haggard, 2003).

### NIBS, Multimodal Integration, Agency and Body Ownership

Empirical investigation of visual-tactile-proprioceptive integration via NIBS globally highlighted the role of inferior parietal areas and higher visual areas for a correct self-attribution of body ownership. The mostly used experimental paradigm to investigate neural and phenomenological correlates of body ownership is the rubber hand illusion—RHI (Botvinick and Cohen, 1998). Each of the few NIBS studies specifically targeting neural basis of body ownership attribution used magnetic stimulation techniques. Namely, in a recent repetitive TMS study, it has been reported that it is possible to strengthen the illusory experience by applying an inhibitory interference to the left extrastriate body area —EBA (Wold et al., 2014). The authors concluded that EBA is involved in multimodal integration for the definition of the experience of body ownership. The body ownership illusion seems, instead, to be weakened by the inhibitory stimulation of the IPL. Low-frequency repetitive stimulation applied to left IPL was indeed found to reduce the relocation of the real hand toward the fake one, even if only immediately after the RHI induction (Kammers et al., 2009). The role of inferior parietal areas—namely, the right temporo-parietal junction, TPJ—was corroborated even by a single-pulse TMS investigation (Tsakiris et al., 2008). By perturbing the activity of right TPJ after brief repeated RHI-inducing stimulations, the authors observed a reduction of the embodiment of the fake hand, as indirectly measured by a reduction of the proprioceptive drift towards the real hand. Processes mediated by inferior parietal areas seem then critical for the definition of the boundaries of our bodies and for the distinction of such objects from external objects and other agents.

## Conclusion

To sum up, NIBS studies mainly focused on the investigation of selected parietal and prefrontal structures, following previous neuroimaging evidences. Nonetheless, a few critical points are worth further discussion.

First, published reports of NIBS investigations are still limited and evidences in literature are fragmented and sometimes contrasting. One of the consequent main limitations has to do with the fact that different NIBS techniques have been used to investigate different function or structures. Physical unwanted effects of TMS are, indeed, usually reported to become more intense and bothering when moving from posterior to anterior areas. tES, instead, can be more comfortably used to stimulate anterior structures since it shows less unwanted effects. Still, the two families of techniques also differ, as above-discussed, in terms of cognitive, spatial, and time resolution. Future research should, then, take advantage of those differences to single out and better understand different facets and features of the agency experience.

Again, NIBS research on agency might benefit from a systematization of methods and measures. Even though the experience of agency pervades our everyday activities, its complex and multi-level nature lead to the development of different elegant experimental design. Nonetheless, a critical discussion on potential indices and measures to qualify and quantify different aspects of sensing agency may help to reduce the fragmentation of empirical findings. Here we began to suggest a potential systematization of empirical findings according to three main facets of research on agency: coding and monitoring of sensorimotor congruence during action, implementation of motor intentions and action control, multimodal integration for self-attribution of body ad action ownership. Such systematization remains valid even if the analysis of present NIBS literature is guided by a criterion concerning experimental methods. Indeed, studies related to the first topic are globally based on visuomotor matching and action attribution paradigms. Studies related to the second topic are essentially based on the intentional binding protocol. Finally, studies related to the third topic are basically based on the RHI protocol. Furthermore, it is worth noting that the vast majority of NIBS investigation of how we sense agency was grounded in indirect and implicit measures of the phenomenon, with no systematic and clear evidence for explicit measures concerning the aware reflexive level of such experience. A systematization might then contribute to find new ways to overcome such methodological limitation and to better understand all the facets of the agency experience, as well as the actual causal role of different cortical structures in relation to both pre-reflexive/implicit and reflexive/explicit agency-related processes.

## Author Contributions

Both DC and MB substantially contributed to the conception of the work and to drafting the work or revising it critically for important intellectual content. Both DC and MB gave their final approval of the version to be published and agreed to be accountable for all aspects of the work in ensuring that questions related to the accuracy or integrity of any part of the work are appropriately investigated and resolved.

## Conflict of Interest Statement

The authors declare that the research was conducted in the absence of any commercial or financial relationships that could be construed as a potential conflict of interest.
